# Editorial

**Published:** 1898-07

**Authors:** 


### Editorial.

### AMERICAN MEDICAL ASSOCIATION.

The meeting at Denver, June 7–10th ultimo, was well attended and up to the standard in scientific work.

In the absence of the President, Dr. George M. Sternberg, the administration of affairs fell to the lot of Vice-President Joseph M. Mathews, of Louisville, who presided with grace and dignity, and so won the hearts of the Fellows as to secure the presidency for the session of 1899. For the second time in a quarter of a century this great honor has been bestowed upon a citizen and surgeon of Louisville, and it is with pride tempered with gratitude that the local guide acknowledges the compliment.

The address of the retiring President was read by Col. A. W. Woodhull, of Denver. It was able, as was expected from a scientist of the first rank, and devoted to the more recent achievements of medicine. The X-ray, the new bacteria, the glandular extracts, antitoxins, and the bearings of these upon prophylaxis and therapy were clearly and forcibly presented. The address from beginning to end was an inspiring testimonial to the recent rapid advancement of medicine upon scientific lines, and heralds the no distant day when medicine, like surgery, shall take its place among the exact sciences, with the rout of quackery and the confusion of fogyism, ignorance, and empty pretension. He thus, in substance, concludes:

Finally, there was no room for creeds and pathies in medicine, no more than in astronomy, geology or botany. Every man was entitled to his own opinions upon any settled problem; but if he entertained an opinion in conflict with ascertained facts he simply showed his ignorance. There was no restriction placed upon any physician who graduates from a regular school as to the mode of treatment he should pursue in a given case. But if his patient dies of diphtheria because of his failure to have administered the proper remedy, or if he recklessly infects a wound with dirty fingers or instruments, or transfers pathogenic streptococci from a case of phlegmonous erysipelas to the interior of the uterus of a puerperal woman, it would appear that the courts should have something to say as to his fitness to practice medicine. There was, however, nothing in the Code of Ethics which should prevent him from associating with reputable practitioners of medicine; but, no matter where or when he obtained his medical degree, he could scarcely be said to belong to the modern school of scientific medicine. We must not fail to recognize, however, that the progress of knowledge has been so rapid that it is impossible for a busy practitioner to keep pace with it, and that even the requirements now generally adopted by our leading medical schools for a four-years' course of study is inadequate for the attainment of such a degree of professional knowledge and practical skill in diagnosis and therapeutics as is desirable for one who intends to practice scientific medicine.

Among the business items the following may be mentioned as pertinent and progressive:

Dr. Dudley S. Reynolds, of Louisville, presented a resolution to the effect that after January 1, 1899, any college professor or other teacher that shall confer any degree upon any person not complying with the standards of the American Medical Association regarding educational requirements, shall hereafter be excluded from meetings of the Association. This was referred to the Executive Committee.

A resolution by Dr. W. W. Keen, of Philadelphia, was also introduced favoring vivisection for experimental research, which was referred to the Executive Committee.

Dr. H. A. Hare, of Philadelphia, offered a resolution relative to the State and county medical societies of New York, asking that they be allowed to send regularly recognized delegates to the Association. Referred to Executive Committee.

Dr. George M. Gould, of Philadelphia, presented a resolution, the effect of which was to encourage the establishment of medical libraries throughout the United States. Referred to Executive Committee.

Dr. Wm. Bailey, of Louisville, introduced an important matter in the form of a resolution to the effect that the office of General Secretary be created, with a salary not to exceed $2,500 or $3,000 per annum. He said that the Association had grown to such proportions that the services of at least one salaried man were required who would devote his entire time to the interests of the Association. By this resolution the present Secretary, Dr. Atkinson, would be retained as an honorary officer. Resolution referred to Executive Committee.

Dr. Sanders, of Alabama, recommended, in a series of resolutions, that the Association take steps leading to the establishment of public health bureaus, having their roots in every city and town in the land, and an executive head in the national government.

Dr. Humiston, of Ohio, read a report, adopted by the Ohio State Medical Society, regarding the anti-vivisection bill, and recommended that in addition to the other committees being named, one should be composed of residents of Washington, Philadelphia, and Baltimore. Under this arrangement there would be assurance of prompt attendance of committeemen whenever the bill referred to came up.

The Committee on Nominations reported: For President, Dr. J. M. Mathews, Louisville; First Vice-President, Dr. W. W. Keen, Philadelphia; Second Vice-President, Dr. J. W. Graham, Denver; Third Vice-President, Dr. H. A. West, Galveston; Fourth Vice-President, Dr. J. E. Minney, Topeka; Librarian, Dr. George W. Webster, Chicago; Trustees, Drs. Alonzo Garcelon, T. J. Happel, and I. N. Love.

Place of meeting, Columbus, Ohio; time, June 7–10, 1899. —*American Practitioner and News.*

---

The Ontario Medical Council has a most interesting and important fight now on. The "great Professor Munyon" of homeopathic-remedy fame some time since opened an office in Toronto, under the management of two regular licensed Canadian practitioners. As is customary with the "Professor" free advice and prescriptions (of Munyon's "Remedies") were advertised extensively in the daily papers, and by circulars. Now it so happens that the legislature of this province has very wisely empowered the Medical Council to erase from the register of qualified practitioners the name of any doctor who violates the common rules of professional etiquette—including such advertising as that of the "Munyon remedies" and their free "prescription," and thereafter such doctor shall not be permitted to practice in the province, regardless of his ability, experience or authority of diploma. So the two doctors in the employ of the Munyon company have been summoned before the Council to show cause why their names shall not be erased from the register. Of course the company has employed the very best legal talent to conduct the fight which will be carried to the highest court in the endeavor to legalize the infamous and disgraceful conduct of the two practitioners and to secure decisions "curtailing the arbitrary and despotic powers" of the Council—to use the expression of the defendants. Attempts are also being made to influence leading newspapers to attack the law and the Council, because under the present rules the extensive advertising which disgraces some papers in the large cities of the United States is not permitted in Ontario; one circular saying: "The success of the council in their war against Munyon will certainly mean an ultimate injury to newspapers in Canada in the loss of advertising." It is to be hoped the Council will ultimately win this fight, which is to be a bitter one.—*American Journal of Surgery and Gynecology.*

